# Effects of Fly Ash Inclusion and Alkali Activation on Physical, Mechanical, and Chemical Properties of Clay

**DOI:** 10.3390/ma15134628

**Published:** 2022-07-01

**Authors:** Canan Turan, Akbar A. Javadi, Raffaele Vinai, Giacomo Russo

**Affiliations:** 1 Department of Engineering, University of Exeter, Exeter EX4 4QF, UK; a.a.javadi@exeter.ac.uk (A.A.J.); r.vinai@exeter.ac.uk (R.V.); 2 Department of Earth Science Environment and Resources, University of Napoli Federico II, DISTAR Building, 80138 Napoli, Italy; giarusso@unina.it

**Keywords:** fly ash, alkali compounds, soil stabilisation, compaction, compressive strength, XRD, SEM

## Abstract

This study investigated the improvement in the behaviour of a clay soil due to the addition of alkali-activated fly ash as a stabilising agent, and the effects of different activation factors such as alkali dosages and silica moduli. The alkali activator solution used was a mixture of sodium silicate and sodium hydroxide. Class F fly ash was used as the precursor material for the geopolymerisation process. Soil samples stabilised with non-activated class F fly ash were prepared and tested to compare the results with samples stabilised with alkali-activated fly ash. Compaction tests, unconfined compressive strength tests, X-ray diffraction analysis, and scanning electron microscopy analysis were carried out on samples cured 1, 7, and 28 days at room conditions. The results showed that the compressive strength of stabilised soil significantly increased when the fly ash was activated. The optimal activation parameters to stabilise the soil were found to be alkali dosages in the range of 12% to 16% and a silica modulus of 1.25. The highest compressive strength recorded was at 1293 kPa with an alkali dosage of 16% and a silica modulus of 1.25, while for the non-stabilised soil, it was at 204 kPa at 28 days of curing. Mineralogical analysis showed a decrease in the peak intensities of kaolinite and illite, while microstructural analysis indicated an alteration in soil texture with the addition of the alkali-activated fly ash.

## 1. Introduction

Due to their low strength and high compressibility, soft clays could experience large deformations under relatively small loads; hence, they could be a significant concern in geotechnical engineering [1,2]. Ordinary Portland cement (OPC) is a common hydraulic binder that has been widely used in geotechnical applications to improve the mechanical properties of soils, such as shear strength and compressibility [3]. However, the production of 1 tonne of OPC releases about 0.7–1.1 tonnes of carbon dioxide [3,4,5,6,7,8]. Producing OPC also releases nitric oxide (NOx), which is one of the factors of the greenhouse effect and acid rain [8,9]. In recent years, geopolymers have gained a lot of attention as alternative materials to use in the construction industry due to being eco-friendly [10]. Geopolymers offer improved mechanical properties and volume stability in soil stabilisation [5], and they are stiff, strong, and durable [8].

Geopolymers can be described as amorphous aluminosilicate cementitious materials [11,12]. They are produced through the reaction of a solid aluminosilicate with an alkali activator and are also termed ‘inorganic polymers’ [13]. Fly ash is one of the waste materials frequently used as a precursor (aluminosilicate-based material) for producing geopolymers, owing to its worldwide availability [9,11,12,14,15,16,17,18,19]. In addition, fly ash contains enough silica and alumina amounts in an amorphous phase which make it an effective precursor for the geopolymerisation process [7,8,14,20]. Geopolymerisation also helps to immobilise the trace toxic metal elements from fly ash, slag, or other industrial wastes that could be used as precursors. The hazardous elements barium (Ba), cadmium (Cd), cobalt (Co), chromium (Cr), and copper (Cu) can be trapped and fixed in the geopolymer matrix. Chemical stabilisation and physical encapsulation are the typical mechanisms of metal immobilisation in the fly ash-based geopolymers [8,12,14,21]. 

In the 1950s, Glukhovsky suggested a model of geopolymerisation separated into three stages; ‘(1) destruction–coagulation; (2) coagulation–condensation; (3) condensation–crystallisation’ [13]. Other researchers extended Glukhovsky’s model to define the geopolymerisation process in detail [13], although studies are still being undertaken to grasp all processes of alkali activation with aluminosilicate materials [22]. The most acceptable geopolymerisation mechanism can be summarised into the following steps:

(1) Dissolution of amorphous aluminosilicate materials from the source material through the action of alkali hydroxide;
(2) Transportation or condensation of precursor ions into monomers;
(3) Polycondensation or polymerisation of monomers into polymers and hardening into geopolymer structure [11,14,23,24].

The three main structures of the geopolymer are:

Poly(sialate) Si:Al = 1(-Si-O-Al-O-);
Poly(sialate-siloxo) Si:Al = 2(-Si-O-Al-O-Si-O-);
Poly(sialate-disiloxo) Si:Al = 3(-Si-O-Al-O-Si-O-Si-O-) [15].

Davidovits [25] offered the name ‘polysialate’, which represents an abbreviation of aluminosilicate oxide. The polysialate/three-dimensional network of aluminosilicate structures can be formulated as:

M_n_ [−(SiO_2_)_z_ − AlO_2_]_n_.wH_2_O
  where M is the alkaline element or cation such as potassium (K^+^), sodium (Na^+^), or calcium (Ca^++^), n is the degree of polycondensation or polymerisation, z is the Si/Al molar ratio, and w is the water content [12,14,26].

The most widely used alkali activators for geopolymerisation are a combination of sodium hydroxide or potassium hydroxide with sodium silicate or potassium silicate [8,27]. The geopolymerisation process is significantly affected by the type of alkali solution used. Studies showed that using a single alkali activator is possible for geopolymerisation; however, when the alkali activator also includes soluble sodium or potassium silicate, the reaction occurs at a faster rate in comparison with using only alkali hydroxides [8,11]. A sodium silicate (SS) solution is generally mixed with a sodium hydroxide (SH) solution to increase compressive strength [12]. In addition, the release of Si^4+^ and Al^3+^ from fly ash in SH solution is higher than that in potassium hydroxide solution [11]. There has been significant research to study the effects of the dosage or concentration of alkali activators with fly ash binders on the mechanical properties of stabilised soil.

Ridtirud et al. [6] performed unconfined compressive strength (UCS) tests on mixtures of class C fly ash and silty clayey gravel with sand (GM-SM) with 1.5%, 3%, 5%, 7%, 10%, 15%, and 20% fly ash (by weight of the soil) at 7, 14, and 28 days of curing. SH solution with a concentration of 8 M was prepared from SH flakes. SS solution had 8.9% of Na_2_O, 28.7% of SiO_3_, 62.5% of H_2_O. The ratios of SH/SS were 1/1, 3/1, 3/2, 2/3, and 1/3. The results indicated that the compressive strength of soil increased with up to 7% fly ash. A possible explanation for this could be that the water and alkali activator solutions were added based on the optimum moisture content; therefore, beyond 7% fly ash, there was not enough solution and water to assist the reaction. The compressive strength of the soil was improved by increasing the curing time. The best ratio of SH/SS solution was found at 1/1 at room temperature, and at 2/3 at 40 °C. 

Trinh and Bui [28] also conducted a series of compressive strength tests on a soil stabilised with activated class F fly ash. An alkali-activated solution (AAS with SS/SH = 2, AAS/fly ash = 0.5) was used. The geopolymer inclusions of 5%, 10%, 15%, and 20% were considered. Sand with different percentages was mixed with clay soil to examine the effects of soil types on compressive strength. It was concluded that the compressive strength of the clay soil increased with the addition of class F fly ash binders and sand contents. 

Correa-Silva et al. [4] examined the effects of alkali-activated low-calcium fly ash through UCS and California bearing ratio (CBR) tests on low-plasticity clayey soil (CL). The ratio of SS/SH solution was 2. UCS and CBR tests were also carried out on samples of the same soil stabilised with cement and lime for comparison with the stabilisation with alkali-activated fly ash. The ratios of fly ash used were 10%, 15%, and 20%, while the ratios of lime and cement were 5%, 7.5%, and 10% and the curing times were 7, 14, 28, and 90 days. According to the UCS test results, the mechanical behaviour of all stabilised soils with 28 days of curing improved compared to unstabilised soil. Fly ash gave significantly better results compared to lime, but the most effective results were obtained with cement at 28 days of curing. Conversely, between 28 and 90 days of curing, the cement improvement was found to be insignificant, whereas lime and fly ash showed a considerable strength increase due to the long-term chemical reactions. The best mechanical behaviour was recorded for the soil stabilised with 20% fly ash at 90 days of curing. Based on the CBR results performed after 96 h, the strength parameters increased by a factor of 6 with addition of 10% of cement, by a factor of 5 with addition of 20% of fly ash, and by a factor of 2.5% with addition of 10% of lime.

Leong et al. [29] analysed the impacts of class F fly ash, alkali activator, water content, curing time, and curing temperature on the mechanical properties of silty sand soil by conducting uniaxial compression strength tests to understand the suitability of such soil stabilised with a fly ash-based geopolymer for the production of bricks. SH or potassium hydroxide and SS were used as the alkali activator. The results showed that the highest compressive strength of the stabilised bricks was found when the ratio of alkali activator/fly ash was at 0.6 and SS/SH or (potassium hydroxide) was at 0.5, with an additional 10% water content. In addition, the compressive strength of the bricks increased by increasing the curing temperature and the ratio of fly ash/soil. On the other hand, the compressive strength of the bricks decreased by increasing the curing temperature above 100° and with an increased curing time. It was, therefore, deduced that the high temperature created cracks on the bricks, resulting in a decrease in compressive strength. The curing days also resulted in a loss of moisture (in the oven) in the bricks. 

Similarly, Dungca and Codilla [30] carried out UCS and CBR tests on class F fly ash-based geopolymer-stabilised silty sand by applying the dry-mix method. In this method, the alkali activators and fly ash were mixed in dry conditions and then water was added. An SS/SH ratio of 2 and an activators-to-fly ash ratio of 0.4 were selected. The curing time was considered 7 days for the CBR and 28 days for the UCS tests. Based on the test results, the obtained UCS values were 78 kPa (medium consistency), 248 kPa (very stiff), and 1350 kPa (hard) with the addition of 10%, 20%, and 30% fly ash-based geopolymer, respectively. The obtained CBR indices were found to be 9.9% and 16.2% (classified as a sub-base material for embankments), and 34.3% (classified as a base material for embankments) with the addition of 10%, 20%, and 30% fly ash-based geopolymer, respectively. 

Sukmak et al. [31] also carried out compressive strength tests to evaluate the effects of fly ash-based geopolymer on the stabilisation of a clay soil. The effects of the fly ash (FA)/clay ratio, SS/SH ratio, and alkali activators (L)/fly ash ratio were considered in the study. The ratios of FA/clay chosen were 0.3, 0.5, and 0.7; the ratios of SS/SH were 0.4, 0.7, and 1, and the ratios of L/FA were 0.4, 0.5, 0.6, and 0.7. Based on the results, the maximum compressive strengths of stabilised soil were found with an SS/SH ratio of 0.7 for all conditions of L/FA and FA/clay ratios. The optimum L/FA ratio was also recommended between 0.5 and 0.6 for the maximum compressive strength. 

The review of the literature describes the mechanical effects of soil stabilisation with different contents of alkali-activated fly ash. It is seen that previous studies reported promising results to improve soil properties with alkali-activated fly ash by conducting essentially UCS and CBR tests. The literature focused on the effects of SS/SH and alkali activator solution/fly ash ratios in these tests. However, SS and SH solutions can be produced and used at different concentrations; thus, data on their ratio alone do not provide enough information for meaningful comparisons among different investigations. In order to provide objective discussions on activator dosages, it is important to focus on the actual contents of Na_2_O and SiO_2_ in the activating solution. Two dosage parameters that have been proposed in this study are the silica modulus (SM), i.e., the mass ratio of SiO_2_/Na_2_O, and the alkali dosage (M+), i.e., the mass ratio of Na_2_O/binder expressed in percentage. Many studies in the geopolymer literature showed the importance of using SM and M+ as the designation of the alkali activators amount, e.g., [7,21,27,32,33,34]. Chi [32] pointed out that the alkali dosages and alkali modulus/silica modulus are two key factors affecting the compressive strength. Karakoc et al. [33] and Gado et al. [24] indicated that the rate of geopolymerisation can be significantly affected by SM ratios, and this geopolymerisation phase has an important effect on the properties and structure of geopolymers. Van Deventer et al. [21] argued that the SM ratio in the activating solutions can significantly change the elemental ratio Si/Al in fly ash-based geopolymers. Yusuf et al. [34] also emphasised that SS solutions produced for the improvement of alkali-activated binders are commercially available with different SM ratios; hence, it is more appropriate and economical to designate optimum SM ratios to obtain the desired strength, rather than only using the SS/SH ratio. 

From the applicability point of view, alkali-activated fly ash is a suitable binder for the stabilisation of marginal geomaterials, an alternative to traditional binders such as lime and/or cement, and has less impact in terms of carbon footprint [35]. It could be applied successfully to enhance the performance of geomaterials since the range of stresses where this improvement technique appears to be effective fits the typical ranges of stresses applied to geotechnical structures well [35].

For the above-mentioned reasons, the literature is quite fragmented when describing the optimal dosages of activators in the field of soil stabilisation. This study systematically investigated the effects of SM and M+ values over the mechanical, physical, and microstructural properties of alkali-activated stabilised clay, offering a novel approach for comparable analysis. The results confirmed the existing literature and expanded the understanding of the effect of fundamental mixing parameters that relates to the chemical nature of the activators.

The aim of the research was to investigate the effects of the binder composition on the compaction and UCS of stabilised soils as well as to assess the mineralogical and microstructural properties of stabilised soil that showed the highest UCS. 

The factors of SM and M+ were used to explain the effects of activator concentration on UCS. Four different silica moduli, four different alkali dosages, and two different fly ash ratios were selected to evaluate the unconfined compressive strength behaviour of alkali-activated fly ash-stabilised soil samples. The effects of SM, M+, curing times, and fly ash dosages on the UCS of the samples were discussed in detail. Before testing UCS, compaction tests were conducted on all samples stabilised with alkali-activated fly ash with different SM and M+ contents to obtain the required optimum moisture content for each sample. The curing times considered were 1, 7, and 28 days for all samples. The tests and analyses indicated above were also conducted on the soil samples stabilised with non-activated fly ash to highlight the effect of alkali activators. The outcome of this research will allow the application of new activating factors, namely, M+ and SM, in soil stabilisation design to obtain the required strength, unlike previously published research, and will foster the use of alkali-activated fly ash as an alternative soil stabiliser, rather than using high-carbon OPC, which gives an advantage in terms of geotechnical engineering applications and environmental aspects. 

## 2. Materials and Methods

### 2.1. Materials

**Figure 1:**
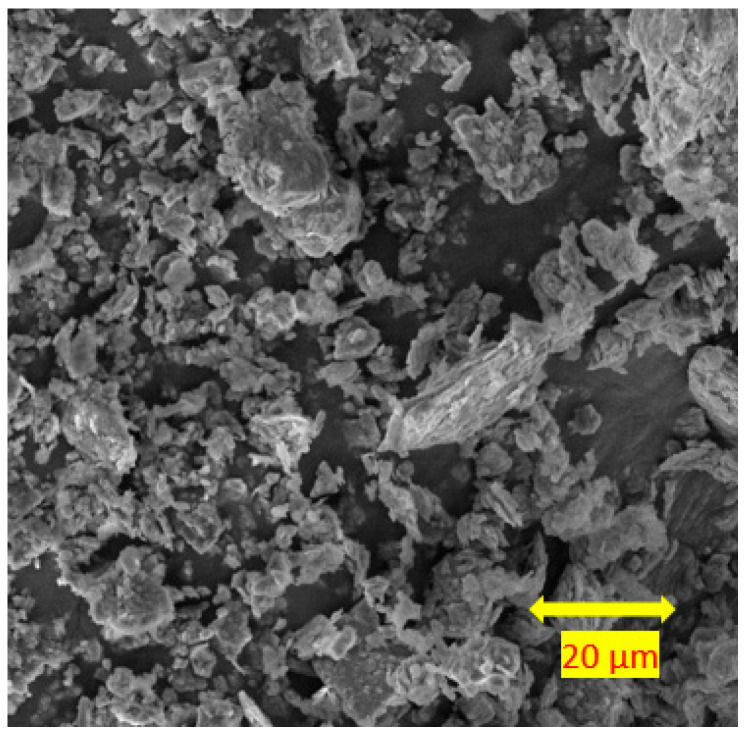
SEM image of kaolinite at 20 μm.

In this study, industrial kaolin clay was chosen in order to avoid adding further variables from the complexities of natural soils and to ensure the repeatability of the experiments. The soil had a liquid limit of 49%, a plastic limit of 25%, and a plasticity index of 24% [36]. According to BS EN ISO 14688-2 [37], the soil was classified as clay with intermediate plasticity (CI). The compaction characteristics of the clay were determined using the standard Proctor test in accordance with BS 1377-4 [38]. The maximum dry density (MDD) and optimum moisture content (OMC) of the clay were 15.2 kN/m^3^ and 21%, respectively. The specific gravity of the clay was determined as 2.6 using the small pycnometer method [36]. SEM images of the clay were taken with a 2.00 kx magnification factor to assess the morphology of the clay. Figure 1 shows that the clay particles were mostly plate-like and irregular in shape. The average oxide compositions of the clay are shown in Table 1.

**Figure 2:**
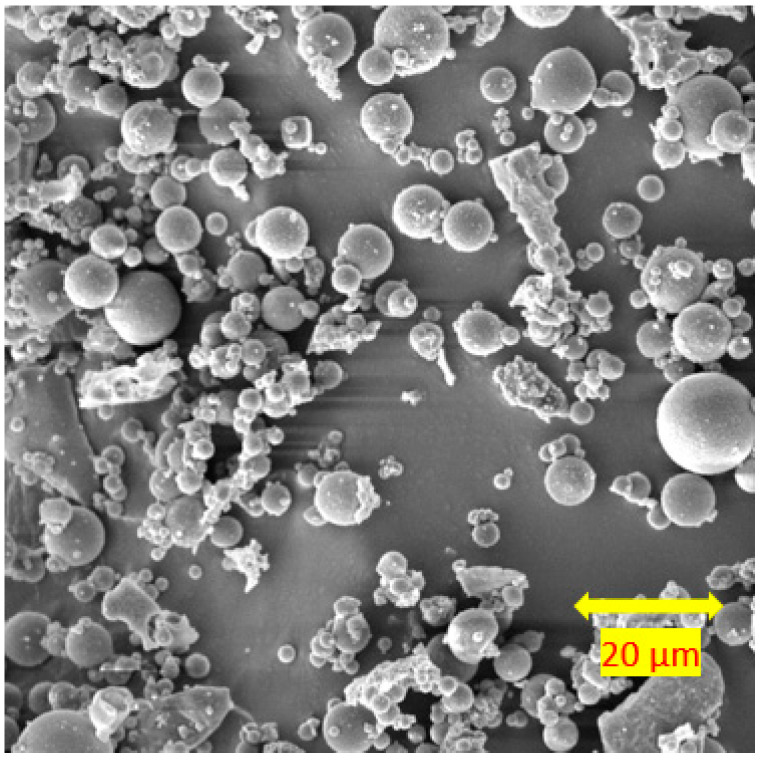
SEM image of fly ash at 20 μm.

Fly ash was acquired from Drax Power Ltd. in the UK. Due to the silt-sized properties of the fly ash, the plasticity index could not be determined; hence, it was noted as non-plastic (NP). The specific gravity of the fly ash was determined as 2.32 from the small pycnometer test. Figure 2 shows the morphology of the fly ash in an SEM image. Based on the SEM analysis, the fly ash had mainly spherical particles. Table 1 shows the average oxide compositions of fly ash. Based on the ASTM C618 [39], the fly ash was classified as class F fly ash. 

Commercially available alkali activators were used in this study. Laboratory-grade SH powder with 99% purity was used. SS solution was supplied from Fisher Scientific Ltd. in the Loughborough, UK, and the mean composition of the solution was as follows: 14% Na_2_O, 28% SiO_2_, and 58% H_2_O, by weight. The mean molecular ratio (SiO_2_: Na_2_O) of the solution was 2.06. 

### 2.2. Sample Preparation

**Figure 3:**
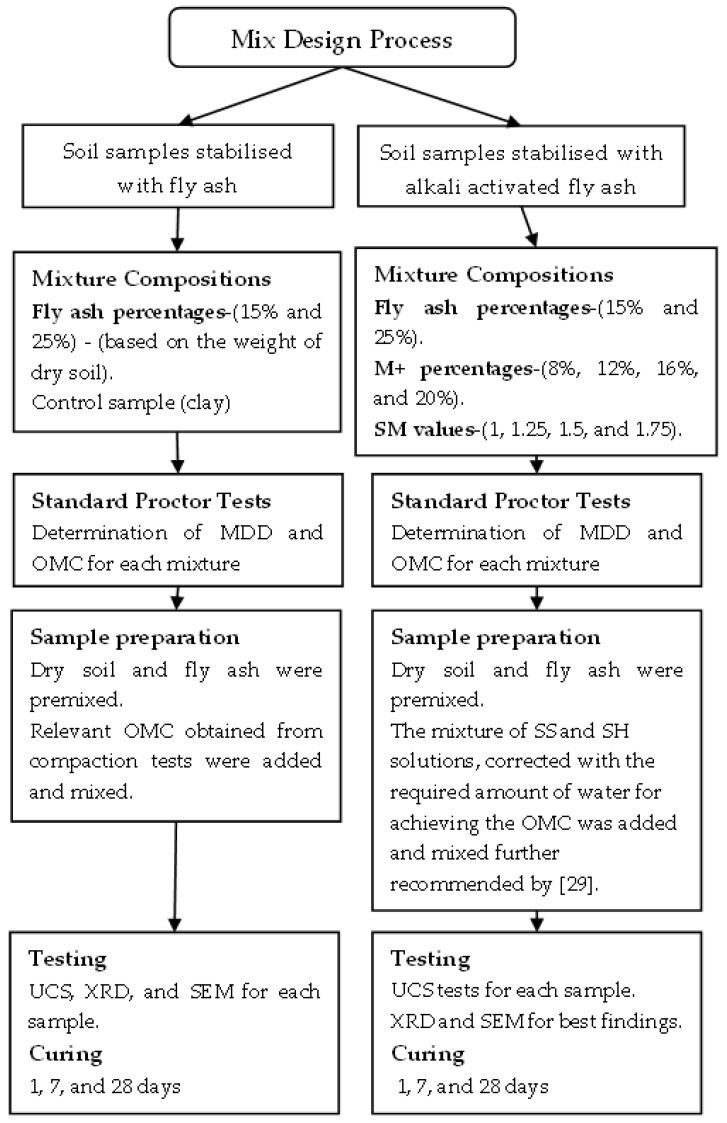
Flow chart of the mix design process.

The general steps of mixture design process are outlined in Figure 3. SM is described as the mass ratio of silica to sodium oxide (Na_2_O) in the activated solution and M+ is the percentage mass ratio of the total Na_2_O in the activated solution to the fly ash binder [40,41]. 

The following steps were followed for the preparation of the alkali activator solution:

- The required amount of Na_2_O was determined based on 8%, 12%, 16%, or 20% of M+ (M+ = Na_2_O/fly ash);
- The required amount of SiO_2_ was evaluated for SM values of 1, 1.25, 1.5, or 1.75 (SM = SiO_2_/Na_2_O);
- The required amount of SS solution was determined based on the required SiO_2_ (SiO_2_ required/SiO_2_ % in solution);
- The chemical composition of SS includes 14% of Na_2_O. Based on this, the required amount of Na_2_O was determined according to the ‘required SS solution’;
- The shortfall of Na_2_O amount in SS solution was completed using SH pellets. Therefore, SH pellets were used to achieve 8%, 12%, 16%, or 20% of M+, considering that the Na_2_O content in SH was 77.5%;
- The required amount of SH pellets was dissolved in distilled water to obtain a solution based on the additional water. The additional water content was determined as:
  additional water = water required (OMC)–water in SS solution;
- Finally, the quantities of SH, SS, and water determined in the previous steps were mixed, and the resulting activating solution was added to the dry soil–fly ash mixture.

### 2.3. Testing Methods

Standard Proctor compaction tests [38] were conducted on control samples, soil samples stabilised with fly ash, and alkali-activated fly ash to obtain their specific compaction characteristics, MDD, and OMC. Abdullah et al. [42] and Mahvash et al. [43] indicated that the delay of compaction in stabilised soil causes early bonds between binders and soil, and these bonds may prevent proper densification of the materials. Therefore, the samples were initially mixed and were thereafter compacted for 30–45 min to prevent the formation of binders in the soil in the loose state [43]. 

UCS tests were conducted on the control samples and soil samples stabilised with fly ash and alkali-activated fly ash, considering different values of SM, M+, percentages of fly ash, and curing times. A longer curing time of more than 1 month might not be practical, specifically for seasonal construction projects. Hence, the conventional 1-, 7-, and 28-day curing methods were considered for the tests. The samples were compacted by static compaction using an Instron (Norwood, MA, USA) 3382 floor model universal testing system. The samples were compacted in three equal layers with a diameter of 50 mm and height of 100 mm, as indicated in [44], were sealed using plastic bags and cured in desiccators at room temperature, and were tested in compression using an Instron 3382 floor model press at a rate of 1 mm/min; the stress–strain curve was obtained for every sample. 

Considering the different combinations of mixtures that were tested, the mixture that provided the best UCS was retained for XRD and SEM analysis. XRD analysis was conducted to obtain the phase composition and mineralogy of the control sample, fly ash, soils stabilised with fly ash, and soils stabilised with alkali-activated fly ash using the Bruker (Billerica, MA, USA) D8 advanced diffractometer. The samples were measured through CuKα rays operated at 40 mA and 40 kV with 2θ range of 5° to 65° in the step size of 0.02°. The XRD patterns were analysed using Diffrac.EVA software with the 2017 reference database. SEM was conducted using TESCAN Vega 3 at the accelerating voltage of 10 kV and the beam intensity of 10 to investigate the microstructural changes of the stabilised soils. Prior to the SEM analysis, the samples were coated using Quorum Q150 TES Sputter Coater with 20 nm thickness and chromium material.

## 3. Results and Discussion

### 3.1. Compaction Tests

**Figure 4:**
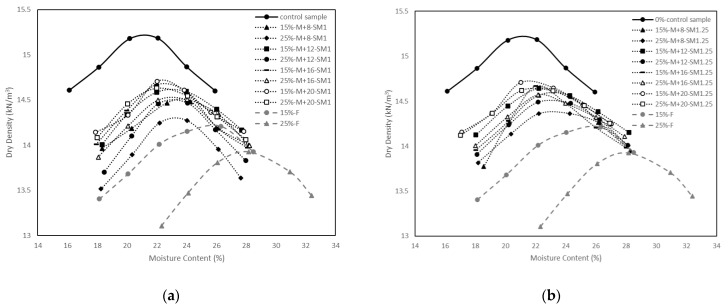

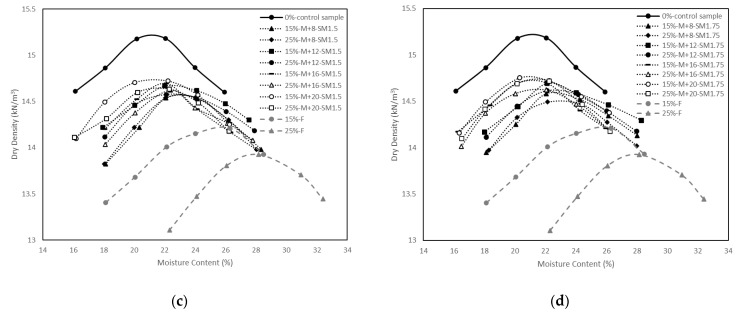
Compaction curves for the control sample, fly ash-stabilised soil samples, and alkali-activated fly ash-stabilised soil samples with different alkali dosages (M+) and silica moduli (SM): (**a**) SM = 1; (**b**) SM = 1.25; (**c**) SM = 1.5; (**d**) SM = 1.75.

Figure 4a–d show the compaction curves of the control sample, stabilised soil samples with different percentages of fly ash, alkali-activated stabilised soil samples with different percentages of fly ash, different M+, and different SM. It is seen that the control sample had higher MDD and lower OMC than the soil stabilised with class F fly ash. A similar trend was reported by Prabakar et al. [45], Mir and Sridharan [46], Seyrek [47]. and Savas [48]. The lower specific gravity of fly ash (2.32) compared to the specific gravity of clay (2.6) could be the reason of the decrease in MDD. According to Murmu et al. [9], the flocculation and agglomeration between clay particles and fly ash lead to an in void ratio, explaining the decrease in MDD with the increase in fly ash. The presence of some broken, hollow fly ash spheres could be the reason for the increase in OMC [46].

Compaction curves of the soil stabilised with alkali-activated fly ash also indicated a decrease in MDD and an increase in OMC in comparison with the control sample. However, the rate of decrease in MDD in soil stabilised with alkali-activated fly ash was less than the soil stabilised with fly ash only. This could be attributed to the alkali activators used in the mixtures being viscous, thereby acting as lubricants among fly ash and clay particles, and resulting in an increase in MDD [49,50]. In addition, it is seen that MDD slightly increased with the increase in M+ and SM for the soils stabilised with alkali-activated fly ash. Although the increase in activator amount seemed to provide an increase in the MDD, the variations were modest. For example, the effect of M+ is indicated in Figure 3, where the MDD was 14.5 kN/m^3^ at M+ of 8%, and it increased to 14.7 kN/m^3^ at M+ of 20% for the soil stabilised with 15% fly ash and an SM of 1. Additionally, MDD was found to be 14.3 kN/m^3^ at M+ of 8%, and it increased to 14.6 kN/m^3^ for the soil stabilised with 25% fly ash and an SM of 1. Similar modest variations in maximum dry density were reported by Sukmak et al. [31], who compared the compaction tests on clay–fly ash and clay–fly ash–geopolymer mixtures. They indicated that the alkali activators insignificantly affect the soil plasticity, and the liquid limit controls the compaction curve. Therefore, the compaction curves of fly ash stabilised soil and alkali-activated fly ash stabilised soil with different binder ratios have similar maximum dry densities for the same fly ash content [31].

The OMC of the soil stabilised with alkali-activated fly ash generally decreased with the increase in M+ and SM. Abdullah et al. [49] argued that the lubrication effects of alkali activators could reduce the required free water to obtain optimum compaction, thereby decreasing the OMC.

### 3.2. Unconfined Compressive Strength (UCS) Tests

Unconfined compressive strength tests were carried out to evaluate the effects of M+, SM, curing time, and fly ash content on the strength of soils stabilised with fly ash and alkali-activated fly ash. The results on the improvement of UCS are summarised in Table 2.

#### 3.2.1. Effects of M+ on UCS

**Figure 5:**
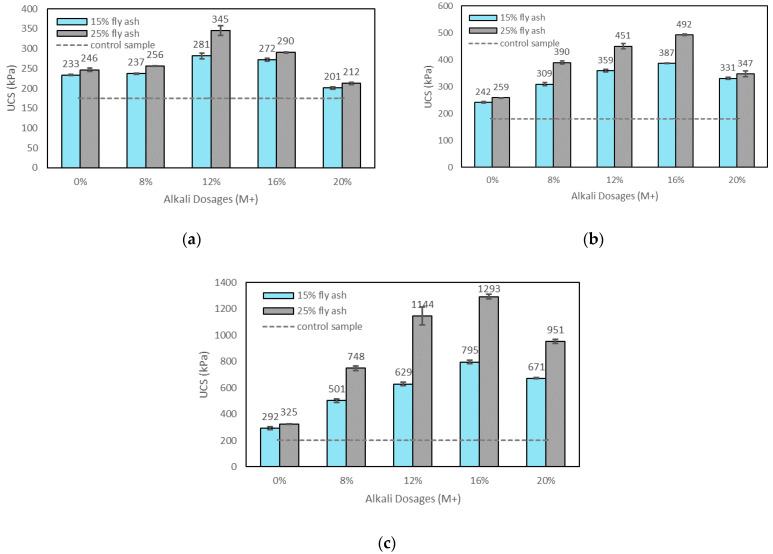
Effects of alkali dosages on the UCS of control sample, soils stabilised with fly ash, and soils stabilised with alkali-activated fly ash with an SM of 1.25 at (**a**) 1 day of curing; (**b**) 7 days of curing; (**c**) 28 days of curing.

M+ mainly represents the alkali concentration in the soil samples. Figure 5a–c show the effects of M+ on the UCS of the control sample, soils stabilised with fly ash, and soils stabilised with alkali-activated fly ash with a constant SM of 1.25 at 1, 7, and 28 days of curing. The results for the soils stabilised with alkali-activated fly ash for different SM values generally showed a similar trend; hence, the best UCS results (SM = 1.25) shown in Figure 5a–c indicate a typical strength improvement of alkali-activated fly ash-stabilised soil with increasing M+ values. It is seen that the compressive strength of the soil samples stabilised with alkali-activated fly ash increased with the increase in M+, up to an optimum value, beyond which a decrease in strength was observed. Alkali dosages (Na_2_O/fly ash) of 8%, 12%, 16%, and 20% were applied as a new designation criterion on soils stabilised with alkali-activated fly ash in this study, while the previous studies applied generally the ratio of alkali activators (SS + SH) solution/fly ash to find the role of alkalinity in the stabilised soils. According to previous studies, the UCS increased with the increase in the ratio of the SS+SH solution/fly ash up to a maximum, then decreased when the activation exceeded the optimal dosages [31,51]. Obtained results are in agreement with this observation. As explained by Phetchuay et al. [51], a higher alkali hydroxide concentration could leach higher Si and Al ions in fly ash for the geopolymerisation reaction and result in a higher compressive strength. Karakoc et al. [33] and Runci and Serdar [52] also indicated that the alkali concentration is a significant factor affecting the geopolymer strength. They reported that an optimum concentration of alkaline accelerated the geopolymerisation and increased the strength. However, when a concentration higher than the optimum was used, a decrease in strength occurred. This is because the excess alkali concentration can precipitate in early stages before polycondensation and hinder the geopolymerisation reaction, reducing the strength [51]. Soutsos et al. [40] also reported that the decrease in strength beyond the optimum M+ value is because the geopolymer gel saturated with alkali ions leads to less free water in the mixture; in this way, the speciation of silica and alumina oligomers from the fly ash dissolution cannot be fully completed with very high M+ values.

In this study, the optimum values of M+ were found to be 16% for 7 and 28 days of curing and 12% for 1 day of curing. The difference is because geopolymerisation is a time-dependent process and the reaction can be completed in the long term. One day of curing would not be enough for the full reaction of the alkali activators. However, with 7 and 28 days of curing, higher amounts of alkali activators will participate in the reaction, increasing the strength of the soil. On the other hand, although the optimum value of M+ was 16% for 7 and 28 days of curing, after the M+ of 12%, the rate of the strength improvement generally decreased (see Figure 5b,c). Hence, the M+ was fixed at 12% for further tests as it ensured satisfactory strength results. Moreover, the M+ of 12% was chosen (rather than 16%) to mitigate the environmental impact of activation chemicals and to reduce cost.

#### 3.2.2. Effects of SM on UCS

**Figure 6:**
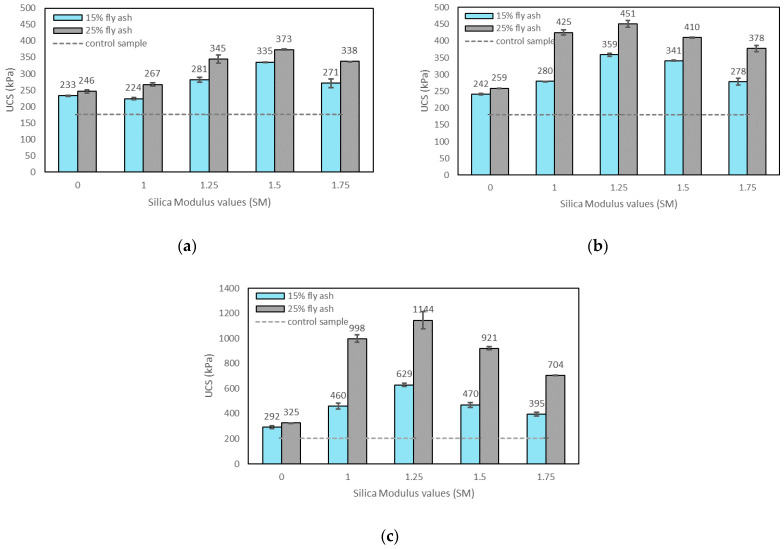
Effects of silica modulus on the UCS of control sample, soils stabilised with fly ash, and soils stabilised with alkali-activated fly ash with an M+ of 12% at (**a**) 1 day of curing; (**b**) 7 days of curing; (**c**) 28 days of curing.

The SM represents the silica amount in the activating solution, and it is the ratio of SiO_2_ to Na_2_O in the solution. Figure 6a–c show the effects of SM on the UCS of the control sample, soils stabilised with fly ash, and soils stabilised with alkali-activated fly ash with a constant M+ of 12% at 1, 7, and 28 days of curing. For an M+ of 8%, 16%, and 20%, the SM trends found are similar. According to the results, an SM of 1.25 gave the highest compressive strength at 7 and 28 days of curing, while the SM of 1.5 was generally found to be the highest at 1 day of curing. The reason for the increase in compressive strength after increasing the SM till 1.25–1.5 could be that the available free Si^+^ in the activating solution aids in the improvement of the polycondensation of oligomeric precursors during the geopolymerisation process. In this way, the degree of geopolymerisation increases, and subsequently, the compressive strength increases [33]. On the other hand, the decrease in the compressive strength after a certain amount of SM is due to the increase in viscosity and the decrease in the pH of the activating solution [7,24]. The increase in sodium silicate content in the activating solution increases viscosity; in this way, the workability of the geopolymer paste decreases [24]. When the SM increases, the alkalinity (pH) of the activating solution decreases due to the decrease in available OH groups, which help the dissolution of materials during the geopolymerisation [24,40].

In general, the silica modulus is inversely proportional to the NaOH and Na_2_O amount in the activating solution. The NaOH participates in the dissolution of aluminosilicates from the reacted fly ash [7,24,29], while free Si^+^ from SS solution is also essential for the development of geopolymer reactions (up to an optimum SM amount) [41]. Therefore, a much higher or lower SM would not result in higher compressive strength, as seen in Figure 6a–c. Although there is a lack of studies using SM on soils stabilised with alkali-activated fly ash, previous studies applied the SS/SH ratio to evaluate the role of SiO_2_ in the geopolymerisation process. Leong et al. [29], Sukmak et al. [31], and Phetchuay et al. [51] used different SS/SH ratios to find the required strength. They indicated that much higher or lower SS/SH ratios in the system are not favourable to balance the SiO_2_ amount. These previous studies are in agreement with the trend of SM observed in this study. 

The SM of 1.25 was found to be adequate to achieve highest strength results with 7 and 28 days of curing. This value was found comparable to the results from Ridtirud et al. [9] and Phetchuay et al. [51], who applied UCS tests on soils stabilised with alkali-activated fly ash with SS/SH ratio. On the other hand, the SM of 1.5 gave the highest UCS at 1 day of curing. The reason for the different optimal SM amount (1.5) with 1 day of curing could be that a higher amount of free Si^+^ is consumed at a faster rate in the short term [24]. However, when the SM is lower (1.25), the OH ions increase and high alkalinity provides better a dissolution of aluminosilicates and gelation, resulting in a better compressive strength in the long term. Phummiphan et al. [15] found that the UCS results were higher with curing time for soil samples with lower SS/SH, which means a lower SiO_2_ and higher NaOH content. According to their results, the highest UCS was at the SS/SH ratio of 90:10 at 28 days of curing, while the SS/SH ratio of 50:50 gave the highest UCS results at curing times longer than 60 days. This is also attributed to the growth of sodium aluminosilicate geopolymer (NASH) gel over time [15].

#### 3.2.3. Effects of Curing Time on UCS

**Figure 7:**
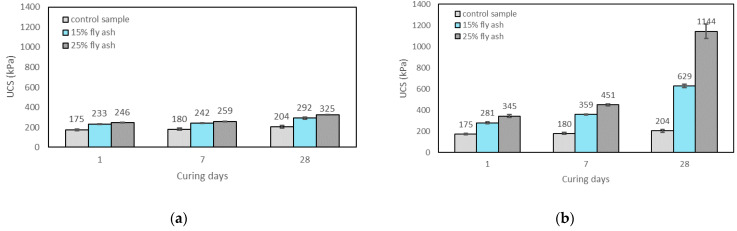

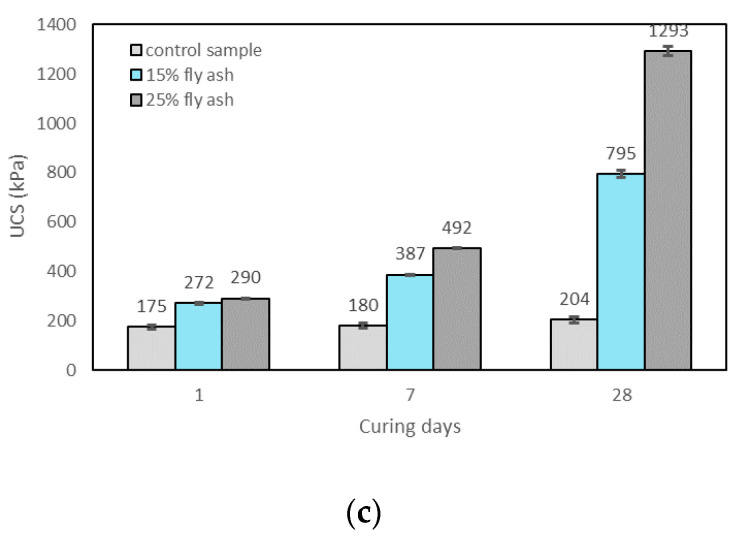
Effects of curing times on the UCS of the control sample: (**a**) soils stabilised with fly ash; (**b**) soils stabilised with alkali-activated fly ash with an M+ of 12% and an SM of 1.25; (**c**) soils stabilised with alkali-activated fly ash with an M+ of 16% and an SM of 1.25.

Figure 7a–c show the variation of UCS with curing time for the control sample, soils stabilised with fly ash, soils stabilised with alkali-activated fly ash with M+ of 12% and SM of 1.25, and soils stabilised with alkali-activated fly ash with M+ of 16% and SM of 1.25, respectively. The increase in UCS with the curing time for the soils stabilised with alkali-activated fly ash with different SM and M+ values generally showed a similar trend (Table 2). The UCS results of the soils stabilised with alkali-activated fly ash showed insignificant improvement at 1 day of curing. This can be attributed to the fact that the alkali activators have low reactivity with Si and Al in fly ash in the initial phase [53]. Parhi et al. [54] argued that the curing time is needed for the reaction to occur and for the products of the reaction derived from the dissolution of Al and Si minerals to accumulate. Sukmak et al. [31] also found that the samples at low temperature or normal room temperature need long curing times to improve the UCS efficiently. The UCS of the soils stabilised with alkali-activated fly ash increased with curing time for different M+ and SM contents, which is in agreement with the previous literature [3,4,15,20,55]. For example, the UCS of the soils stabilised with 15% alkali-activated fly ash with an M+ of 12% and an SM of 1.25 cured for 1, 7, and 28 days were found to be 1.6, 2, and 3.1 times that of the control sample, respectively. In addition, the UCS results of soils stabilised with 25% alkali-activated fly ash for an M+ of 12% and an SM of 1.25 cured for 1, 7, and 28 days were 2, 2.5, and 5.6 times that of the control sample. The highest improvement was observed in soils stabilised with 25% alkali-activated fly ash for an M+ of 16% and an SM of 1.25 cured for 28 days, with the UCS increasing by 6.3 times that of the control sample (Figure 7c). The improvement in UCS with the curing times is attributed to the continuity of the geopolymerisation reaction [55]. The reactions between fly ash, SS, and SH lead to the NASH products [15]. Due to the time-dependent availability of NASH products, the UCS of samples increases with the increase in curing time, with the presence of SH. On the other hand, the soils stabilised with neat fly ash showed a very slight increase with the increase in curing time (Figure 7a). The increase in UCS in soils stabilised with fly ash with the curing time is due to the pozzolanic properties of fly ash [56]. 

**Figure 8:**
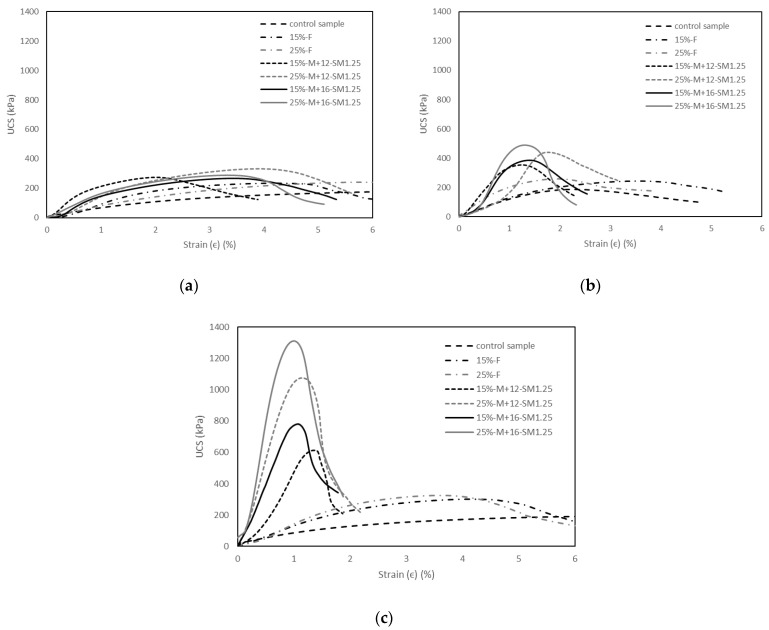
Stress-strain behaviour of the control sample, soils stabilised with 15% and 25% of fly ash, and soils stabilised with 15% and 25% of alkali-activated fly ash with a constant SM of 1.25 and an M+ of 12% or 16% at different curing times: (**a**) 1 day of curing; (**b**) 7 days of curing; (**c**) 28 days of curing.

Figure 8a–c show the stress–strain behaviour of the control sample, soils stabilised with 15% and 25% fly ash, and soils stabilised with 15% and 25% alkali-activated fly ash with a constant SM of 1.25 and an M+ of 12% and an M+ of 16%, cured for 1, 7, and 28 days. The stress–strain behaviour of the soils stabilised with alkali-activated fly ash for different SM and M+ values generally showed a similar trend. It is seen that the control sample and soils stabilised with fly ash generally showed a ductile behaviour at different curing times. Moreover, the soils stabilised with alkali-activated fly ash at 1 day of curing showed a similar ductile response. This is because the geopolymerisation at 1 day of curing is in the initial phase due to the relatively low reactivity of the system. With the increase in curing time, the stress–strain behaviour of the soils stabilised with alkali-activated fly ash changed from a ductile to a brittle response at 7 and 28 days of curing. The soils stabilised with alkali-activated fly ash at 7 and 28 days of curing indicated higher initial stiffnesses, followed by a sudden strain-softening behaviour. For example, the peak UCS of the control sample was 190.8 kPa at the axial strain of 6%, whereas the peak UCS of the soil stabilised with 25% alkali-activated fly ash with an SM of 1.25 and an M+ of 16% was 1312.3 kPa at the axial strain of 1% and 28 days of curing. A similar trend was reported in previous studies, and the improvement in the stress–strain behaviour of soils stabilised with alkali-activated fly ash was attributed to the formation of cementitious NASH products [55]. Essentially, the cementitious products bonded and increased the strength of the soil structure, leading to brittle and stiff bridges in the stabilised soil; hence, the bonds were destructured at very low strain. Kamruzzaman et al. [57] also indicated that the sudden strain-softening behaviour post yield is because the more the cementitious the bonds, the higher the destructuration in the soil matrix. 

#### 3.2.4. Effects of Fly Ash on UCS

**Figure 9:**
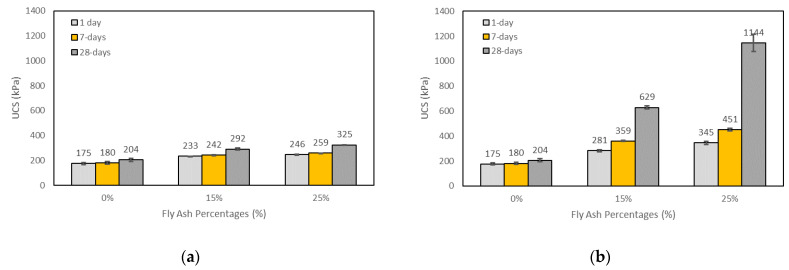
Effects of fly ash content on the UCS of (**a**) soils stabilised with neat fly ash and (**b**) soils stabilised with alkali-activated fly ash with an M+ of 12% and an SM of 1.25 and with different curing times.

Figure 9a,b show typical results of the effects of fly ash content on the UCS of soils stabilised with fly ash and with alkali-activated fly ash with the constant M+ of 12% and SM of 1.25 at 1, 7, and 28 days of curing. It can be seen that the UCS of the soils stabilised with fly ash or alkali-activated fly ash increased with the increase in fly ash content. This is in agreement with the previous investigations [3,29,31,51]. However, the results for the soils stabilised with alkali-activated fly ash at 1 day of curing, and for the soils stabilised with fly ash at 1, 7, and 28 days of curing, were found to be insignificant. This might be due to the fact that class F fly ash has very low reactive calcium content, and this results in a lack of a chemical reaction with soil [56]. On the other hand, the UCS of the soils stabilised with alkali-activated fly ash increased considerably with the increase in fly ash content at 7 and 28 days of curing. In general, the UCS of the soils stabilised with alkali-activated fly ash almost doubled, from 15% to 25% fly ash content, at 28 days of curing. For example, when the fly ash content increased from 15% to 25%, the UCS of the soils stabilised with alkali-activated fly ash for the SM of 1.25 and M+ of 12% increased from 281 kPa to 345 kPa, from 359 kPa to 451 kPa, and from 629 kPa to 1144 kPa at 1, 7, and 28 days of curing, respectively. The reason for the increase in the UCS with the increase in the fly ash content in the stabilised soil is that the fly ash includes SiO_2_ and Al_2_O_3_ in the amorphous phase; hence, it can react effectively with SS and SH. In this way, more geopolymer can be formed to bind with soil particles due to the higher consumed SiO_2_ and Al_2_O_3_, leading to higher compressive strengths [29,51]. Sukmak et al. [31] showed, from the results of X-ray fluorescence (XRF) analysis, that the fly ash is mostly in the amorphous phase; therefore, the leaching capacity of SiO_2_ and Al_2_O_3_ is high. They also indicated that clay includes negative aluminosilicate layer surfaces, resulting in high cation exchange capacity (CEC). As these negative surfaces have high anion capacity, they swarm positive cations (e.g., Na, K). When the fly ash content increases, the negative surfaces in the mixture decrease. In this way, both the increase in amorphous aluminosilicate and the decrease in negative surfaces lead to an increase in the geopolymerisation degree [31]. 

### 3.3. X-ray Diffraction Analysis

**Figure 10:**
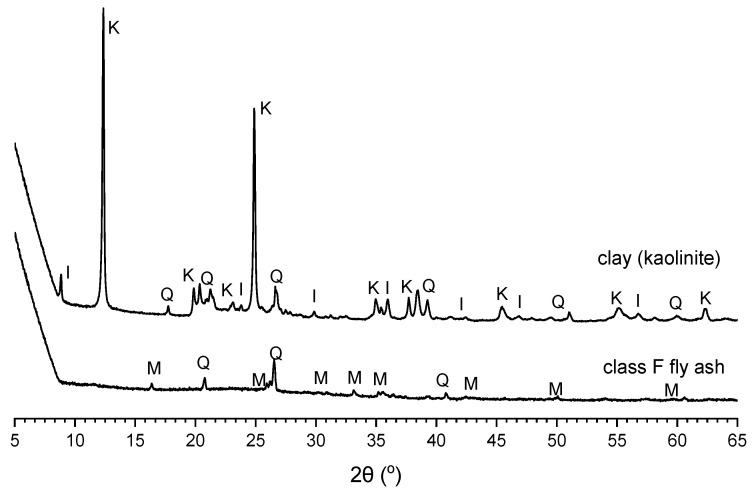
XRD patterns of clay and class F fly ash; I = illite, K = kaolinite, Q = quartz, M = mullite.

The XRD patterns of the control sample (clay) and class F fly ash are shown in Figure 10. The clay was mainly composed of crystalline kaolinite, illite, and quartz, while the fly ash showed mostly amorphous phase with some crystalline mullite and quartz.

**Figure 11:**
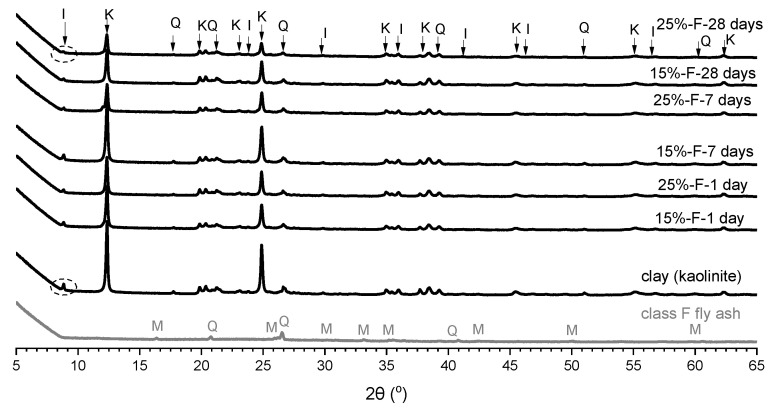
XRD patterns of fly ash, clay, and soils stabilised with 15% and 25% of class F fly ash at 1 day, 7 days, and 28 days of curing; I = illite, K = kaolinite, Q = quartz.

Figure 11 shows the XRD patterns for fly ash, clay, and soils stabilised with 15% and 25% fly ash at different curing times. These patterns are used to analyse the change in mineralogy and crystalline structure. It is seen that there was no mineralogical change in the XRD patterns for the soil stabilised with neat fly ash. However, the intensity of the sharp peaks detected at 2θ values of about 12° and 25° in crystalline kaolinite and 2θ value of 9° in illite slightly decreased when increasing the curing time and fly ash content. Additionally, the peaks of quartz detected at 2θ values of 19° and 52° generally showed small changes with the curing time. Sun and Vollpracht [58] indicated that the dissolution of minerals would occur under a high pH environment. Therefore, for soils stabilised with fly ash, the dissolution of kaolinite and illite may not be possible due to the low pH environment. Moreover, the dissolution of quartz is not possible since it is an inert mineral [59]. Thus, the slight decrease in the peak when the kaolinite is mixed with fly ash may be explained by the fact that the minerals become less concentrated due to dilution. The low amount of clay minerals in the peak intensities could support the decrease in the swelling properties of the clay soil [54]. The lack of mineralogical change in the XRD patterns for the soil stabilised with fly ash confirmed that no chemical reaction between clay minerals and class F fly ash occurred.

**Figure 12:**
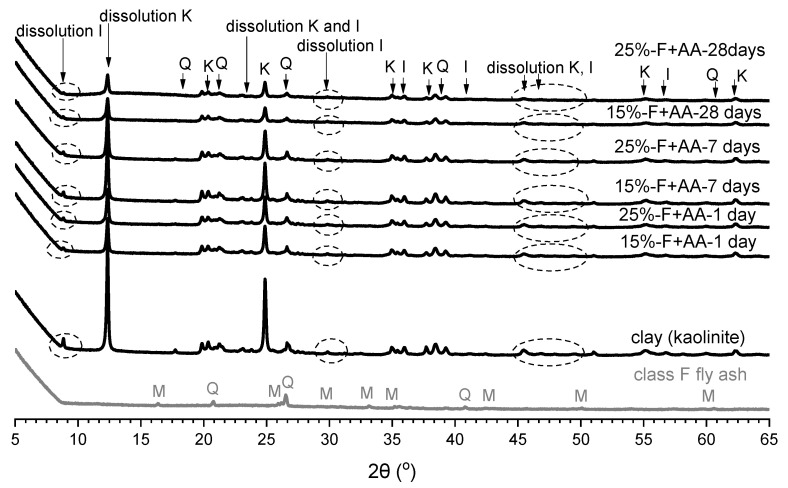
XRD patterns of fly ash, clay, and soil stabilised with 15% and 25% of alkali-activated fly ash at 1 day, 7 days, and 28 days of curing; I = illite, K = kaolinite, Q = quartz.

**Figure 13:**
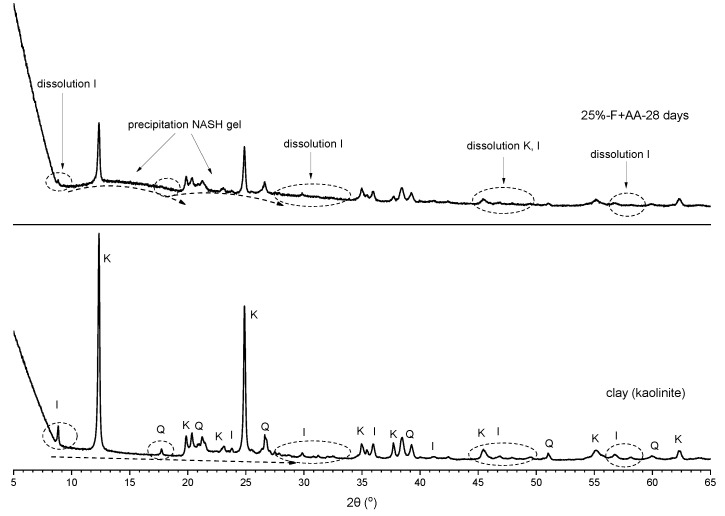
XRD patterns of clay and soil stabilised with 25% of alkali-activated fly ash at 28 days of curing; I = illite, K = kaolinite, Q = quartz.

Figure 12 shows the XRD patterns for fly ash, clay, and soil stabilised with alkali-activated fly ash for M+ of 12% and SM of 1.25 at different curing times. A significant decrease in the peak intensities of kaolinite and illite was observed in the stabilised soil at each curing time. Essentially, illite detected at 2θ values of 9°, 24°, and 47° was fully dissolved in the soil stabilised with 15% or 25% of alkali-activated fly ash at 28 days of curing. The dissolution of minerals in clay and the dissolution of fly ash leads to the formation of amorphous phase in the stabilised soil. This amorphous phase was observed as a broad hump in the XRD pattern detected at 2θ values of between 12° and 28° at 28 days of curing, as shown in Figure 13. This ‘featureless hump’ indicated the newly precipitated compounds due to the geopolymerisation as described by Duxson et al. [13]. The new amorphous geopolymer phase detected at 2θ values of 12–28° indicated the formation of NASH gel [53,60,61,62,63]. On the other hand, no new crystalline phase was observed in the stabilised soil. Duxson et al. [13] argued that the transition from amorphous to crystalline phases of geopolymers is significantly affected by temperature, aging, and soluble silica amount. Based on their study, no new crystalline phases were observed with curing at 70 °C or 90 °C, whereas curing at 120 °C showed new crystalline phases. In addition, high level of soluble silica in the samples represented more amorphous and featureless humps in XRD analysis [13].

In general, the soil stabilised with neat fly ash presented a slight decrease in the peaks of mainly kaolinite and illite during curing. On the other hand, the soil stabilised with alkali-activated fly ash presented high dissolution starting from 1 day to 28 days of curing time. The dissolution of kaolinite and illite, followed by the formation of NASH gel, could correspond to the fewer swelling properties and higher UCS in the soil stabilised with alkali-activated fly ash. 

### 3.4. Scanning Electron Microscopy

**Figure 14:**
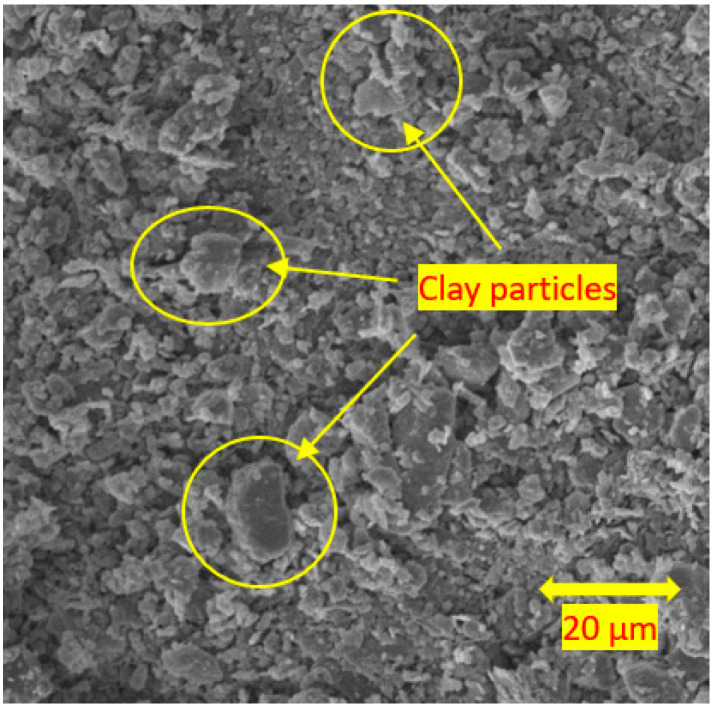
SEM image of clay at 20 μm.

**Figure 15:**
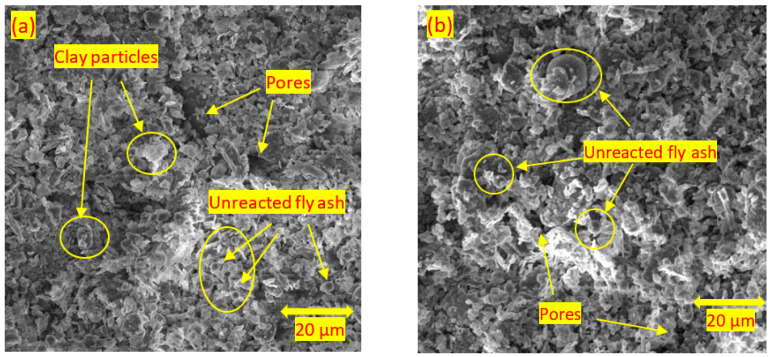

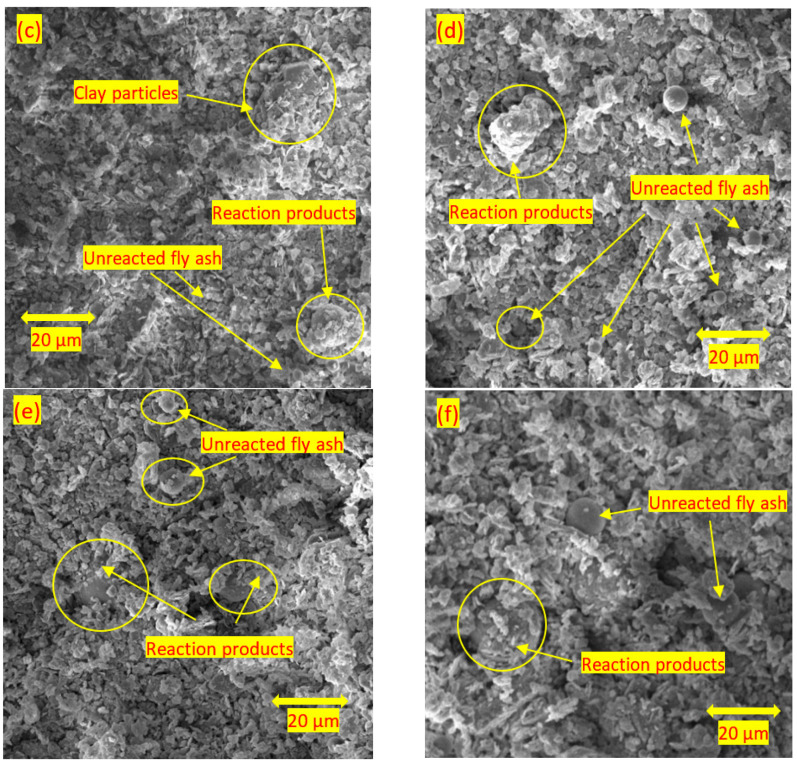
SEM images of soils stabilised with (**a**) 15% fly ash at 1 day of curing; (**b**) 25% fly ash at 1 day of curing; (**c**) 15% fly ash at 7 days of curing; (**d**) 25% fly ash at 7 days of curing; (**e**) 15% fly ash at 28 days of curing; and (**f**) 25% fly ash at 28 days of curing at 20 μm.

**Figure 16:**
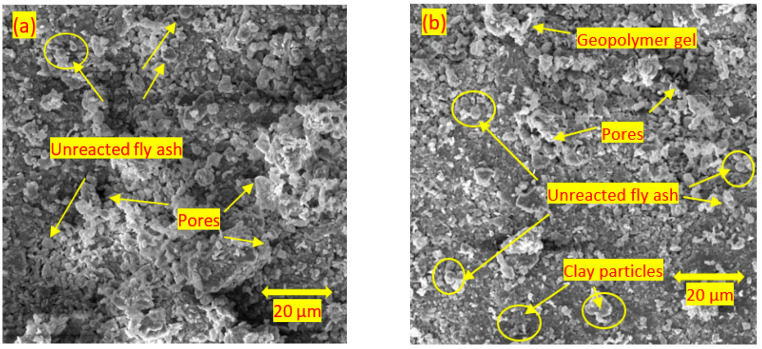

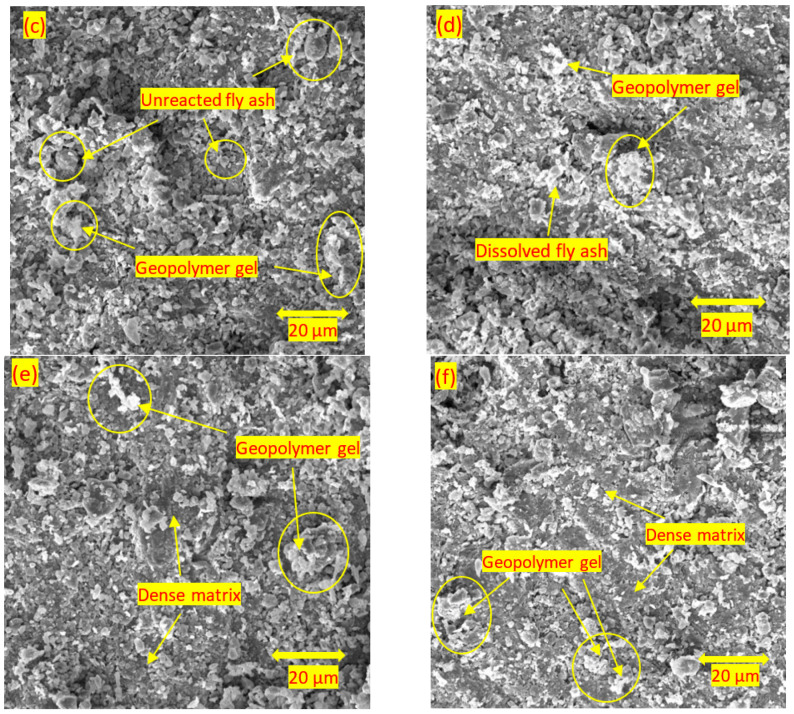
SEM images of soils stabilised with (**a**) 15% of alkali-activated fly ash at 1 day of curing; (**b**) 25% of alkali-activated fly ash at 1 day of curing; (**c**) 15% of alkali-activated fly ash at 7 days of curing; (**d**) 25% of alkali-activated fly ash at 7 days of curing; (**e**) 15% of alkali-activated fly ash at 28 days of curing; and (**f**) 25% of alkali-activated fly ash at 28 days of curing at 20 μm.

The microstructures of the control sample, the soils stabilised with fly ash, and the soils stabilised with alkali-activated fly ash samples (M+ of 12% and SM of 1.25) are shown in Figure 14, Figure 15 and Figure 16, respectively. Aggregated samples were used to observe the entire microstructure of the soil. The figures show the SEM images that are magnified to 2.00 kx. Figure 14 shows the plate-like particles of the non-stabilised clay that was also reported by Abdullah et al. [64]. Generally, a regular microstructure was observed in the clay morphology. 

Figure 15a–f show the results of the SEM analysis on the soils stabilised with neat class F fly ash. Unreacted fly ash particles were observed in the stabilised soils at each curing time. This is because class F fly ash does not react in the absence of a suitable, high pH chemical environment. On the other hand, the presence of reaction products was observed at 7 and 28 days of curing due to the pozzolanic reactions (Figure 15c–f). Reaction products were observed due to enhanced pozzolanic reactions in SEM images, similar to those reported by Raj et al. [65]. Even though the reaction products observed in the system improved the mechanical properties of stabilised soil, the presence of unreacted fly ash at 28 days of curing showed the insignificant improvement in the soils stabilised with class F fly ash. Turan et al. [56] indicated that due to the low Ca content in class F fly ash, it has a lack of cementitious properties; hence, class F fly ash is not able to give a strong reaction with soil. 

Figure 16a,b show the results for the soils stabilised with 15% and 25% of alkali-activated fly ash after 1 day of curing. The structure of the samples predominantly consisted of pores, hollow cavities, and unreacted/partially dissolved fly ash cenospheres. This is in agreement with the findings of Jaditager and Sivakugan [66] and Syed et al. [53], who found pores in the stabilised soil at an early age. NASH cementitious products or geopolymer gels were also observed around the fly ash and clay particles in several parts of the samples; however, more cementitious products were formed with the increase in the fly ash content. Rios et al. [20] and Abdullah et al. [64] argued that aluminosilicate materials from the fly ash leach because of the highly alkaline environment in activators; hence, the spherical shape of some ash particles disappears due to the dissolution process. Thereafter, NASH cementitious products coat clay particles and the remaining fly ash particles. Figure 16c,d show the results for 7 days of curing for the soil stabilised with 15% and 25% of alkali-activated fly ash. The morphologies of the samples were dominated by structured/aggregated clay particles connected by NASH geopolymer gels and unreacted/partially dissolved fly ash particles. Figure 16e,f show the results for 28 days of curing for the soil stabilised with 15% and 25% of alkali-activated fly ash. The samples were found to have a dense matrix structure. During the curing time, pores and hollow cavities were filled with cementitious products/geopolymer gels and a dense matrix was formed. This finding is in agreement with those observed in the previous studies on soils stabilised with alkali-activated fly ash [3,15,53]. Lin et al. [67] also indicated that the denser the microstructure, the higher the compressive strength, which is comparable with the findings in Section 3.2.

In general, the soils stabilised with fly ash did not present the significant improvement in the soil morphology, whereas by increasing the alkali-activated fly ash dosage and curing time, the samples showed a denser structure and fewer unreacted fly ash particles. The microstructural studies conducted on the soil stabilised with alkali-activated fly ash showed a reinforcement in the clay structure due to the formation of cementitious products during curing, which led to an improvement in compressive strength. In addition, the pores and hollow cavities found at the early curing time were filled with the cementitious products. The UCS results obtained in this study are in good agreement with the SEM analysis findings.

## 4. Conclusions

This study investigated the effects of class F fly ash and class F fly ash + alkali activators (SH and SS) as stabilisation additives on the compaction, strength, mineralogical, and microstructural behaviour of clay soil at different curing times. The following conclusions can be drawn from the results obtained in this study:

- The control sample had higher MDD and lower OMC than the soil stabilised with fly ash and the soil stabilised with alkali-activated fly ash. The modest increase in MDD ranging from 14.3 to 14.7 kN/m^3^ and the modest decrease in OMC ranging from 23.8 to 21.6% were observed with the increase in M+ and SM;
- The UCS of the soil increased with the addition of alkali activators, fly ash content, and the curing time. The highest UCS result was found with 25% fly ash content, M+ of 16% and SM of 1.25 at 28 days of curing which can improve the UCS by about 6.3 times over the control sample;
- M+ = 12% was found to be optimal at 1 day of curing, whereas M+ = 16% gave the highest strengths at 7 and 28 days of curing. However, the increase rate of UCS from M+ of 12% to M+ of 16% was generally found to be marginal; thus M+ = 12% was chosen for further tests;
- SM = 1.25 resulted in the highest strength improvement at 7 and 28 days of curing, and was therefore chosen for further tests;
- The XRD analysis showed that, in the soil stabilised with fly ash, the peak intensities of kaolinite and illite slightly decreased at 28 days of curing, whereas in the soil stabilised with alkali-activated fly ash, the peak intensities of kaolinite and illite significantly decreased at all curing times;
- For the soils stabilised with alkali-activated fly ash, the microstructure of the soil was altered with the addition of alkali-activated fly ash, resulting in pores and hollow cavities at an early age. During the curing, aggregated-coarser particles were observed, leading to a denser fabric. On the other hand, the soils stabilised with fly ash showed insignificant microstructural behaviour.

In general, it was observed that stabilisation with alkali-activated fly ash had significantly positive effects on the mechanical properties of the clay soil, and it would be replaced with ordinary Portland cement to mitigate environmental impacts. Further studies on the environmental impacts and a durability assessment are recommended to understand the applicability of soils stabilised with alkali-activated fly ash. 

## Figures and Tables

**Table 1:** Oxide compositions of clay and class F fly ash.

Chemical Composition	Clay	Fly Ash
CaO (%)	0.1	2.2
SiO_2_ (%)	54.8	48.6
Al_2_O_3_ (%)	41.1	22.5
Fe_2_O_3_ (%)	1.0	9.2
K_2_O (%)	2.1	4.1
MgO (%)	0.4	1.3
Na_2_O (%)	0.1	0.9
P_2_O_5_ (%)	0.1	0.2
SO_3_ (%)	-	0.9
TiO_2_ (%)	0.1	1.1

**Table 2:** UCS in kPa of soils stabilised with fly ash and with alkali-activated fly ash with different alkali dosages and silica moduli.

		1 Day Curing		7 Days Curing		28 Days Curing	
				Fly Ash Content		Fly Ash Content	
M+	SM	15%	25%	15%	25%	15%	25%
0%	0	233	246	242	259	292	325
8%	1	210	253	255	373	422	687
	1.25	237	256	309	390	501	748
	1.5	244	276	262	385	413	621
	1.75	230	263	236	311	384	504
12%	1	224	267	280	425	460	998
	1.25	281	345	359	451	629	1144
	1.5	335	373	341	410	470	921
	1.75	271	338	278	378	395	704
16%	1	224	265	306	472	566	1106
	1.25	272	290	387	492	795	1293
	1.5	305	335	342	483	539	1097
	1.75	281	297	313	436	485	838
20%	1	172	201	269	296	441	834
	1.25	201	212	331	347	671	951
	1.5	208	219	277	298	404	782
	1.75	172	197	247	284	352	677

## Data Availability

Some of the data used to support the findings of this study can be made available from the corresponding author upon request.
